# Enhancing Schistosomiasis Control Strategy for Zimbabwe: Building on Past Experiences

**DOI:** 10.1155/2012/353768

**Published:** 2012-05-08

**Authors:** Moses J. Chimbari

**Affiliations:** Okavango Research Institute, University of Botswana, P. Bag 285, Maun, Botswana

## Abstract

*Schistosoma haematobium* and *Schistosoma mansoni* are prevalent in Zimbabwe to levels that make schistosomiasis a public health problem. Following three national surveys to map the disease prevalence, a national policy on control of schistosomiasis and soil transmitted helminths is being developed. This paper reviews the experiences that Zimbabwe has in the area of schistosomiasis control with a view to influence policy. A case study approach to highlight key experiences and outcomes was adopted. The benefits derived from intersectoral collaboration that led to the development of a model irrigation scheme that incorporates schistosomiasis control measures are highlighted. Similarly, the benefits of using plant molluscicides and fish and duck biological agents (*Sargochromis codringtonii* and *Cairina moschata*) are highlighted. Emphasis was also placed on the importance of utilizing locally developed water and sanitation technologies and the critical human resource base in the area of schistosomiasis developed over years. After synthesis of the case studies presented, it was concluded that while there is a need to follow the WHO recommended guidelines for schistosomiasis control it is important to develop a control strategy that is informed by work already done in the country. The importance of having a policy and local guidelines for schistosomiasis control is emphasized.

## 1. Introduction

**Figure 1:**
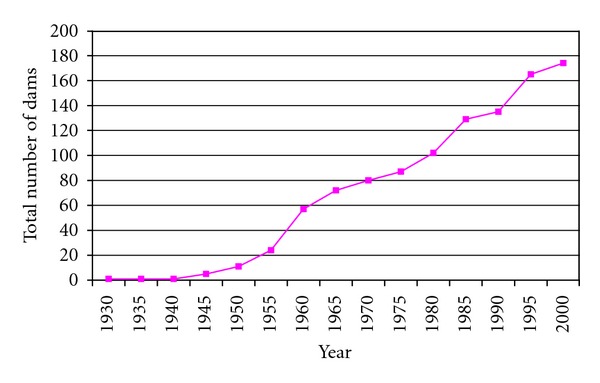
Cumulative number of small dams in Zimbabwe (1930–2000) Adapted from Senzanje and Chimbari (2002). Inventory of small dams in Africa: A case study for Zimbabwe.

Schistosomiasis has, for many decades, been among the top ten causes of hospital admissions in Zimbabwe, an indication of its public health importance [1]. Before the advent of HIV and AIDS, the disease ranked second after malaria in terms of public health importance. *Schistosoma haematobium* and *S. mansoni* are prevalent countrywide and their epidemiology has been studied extensively [2–6] Apart from site specific prevalence and incidence studies [7–11], three national surveys have been conducted since 1982 [2, 3]. Ndhlovu et al. [3] reported that *S. haematobium* was more widely distributed in Matabeleland South province than previously reported by Taylor and Makura [4]. The authors [3] also reported presence of *S. mansoni* in areas where it was not previously reported. Ndhlovu et al. [3] attributed the observed differences in distribution and prevalence of schistosomiasis to increased dam projects in the provinces (Figure 1) and population movements following the country's independence from colonial rule as well as a laxity in schistosomiasis control activities. The most recent survey [2] has shown that schistosomiasis is still prevalent in Zimbabwe with overall *S. haematobium* and *S. mansoni* prevalences of 20.8% and 9%, respectively.

Given the more thorough approach taken in the latest national survey [2], future efforts to control schistosomiasis in Zimbabwe should be informed by results of that survey. It should, however, be noted that because of the global shift from an integrated approach to schistosomiasis control that included control of intermediate host snails to a treatment based approach [12], the most recent survey did not include snail aspects. Nonetheless, a good overview of the distribution of the two intermediate hosts in Zimbabwe (*Bulinus globosus* and *Biomphalaria pfeifferi*) for schistosomiasis is known from previous studies [13].

On the basis of the most recent national schistosomiasis survey [2], a national control policy for Zimbabwe was drafted and will soon go through the necessary national structures responsible for policy formulation. At the policy formulation workshop, where the draft policy document was drafted, the evidence of successes made in controlling schistosomiasis through inclusion of other strategies apart from chemotherapy was highlighted and inclusion of such strategies in the policy was proposed.

This paper reviews schistosomiasis control activities that have been conducted in Zimbabwe over the years, highlighting key lessons that may be applied to develop a home grown strategy for controlling schistosomiasis in Zimbabwe.

## 2. Methodology

**Figure 2:**
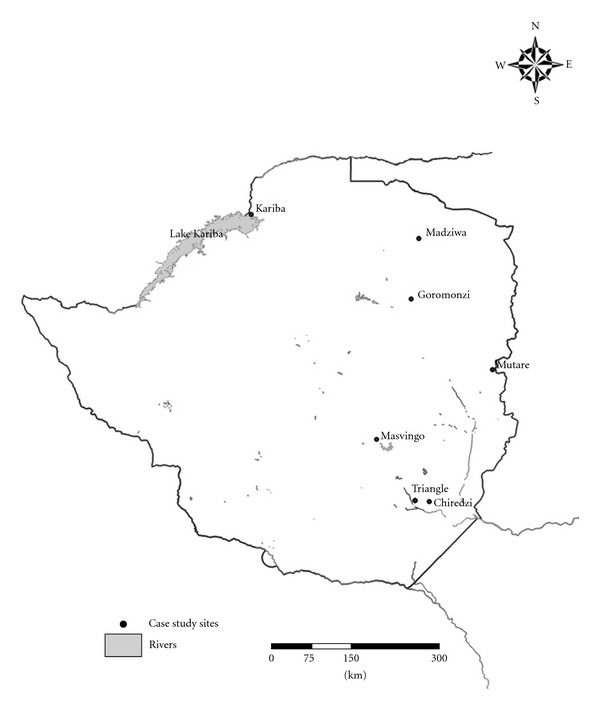
Map of Zimbabwe showing locations of case studies reviewed *(produced by Mrs. A. Makati, GIS Laboratory, Okavango Research Institute). *

This paper is based on case studies on schistosomiasis control activities in Zimbabwe, and all the work presented has been published elsewhere or exists as grey literature mainly in project reports available at the National Institute of Health Research (formerly Blair Research Laboratory), Harare Zimbabwe. Although much work on schistosomiasis control has been done in Zimbabwe since 1960s, this paper focuses on key case studies that made significant impact on the prevalences of the two parasites and therefore should be used as lessons and should inform the proposed national schistosomiasis control strategy/policy. Some of the cases are in the form of research projects and intervention trials, while others are robust control interventions implemented over protracted periods of time.  Figure 2 shows the locations of the case studies reviewed.

## 3. Case Studies on Zimbabwe Schistosomiasis Control Experiences

The case studies described in this paper highlight the experience that Zimbabwe has regarding alternative control strategies for schistosomiasis. The case studies are as follows: (i) Kariba Dam schistosomias is control programme, (ii) Mushandike schistosomiasis control programme, (iii) Hippo Valley Sugar Estates schistosomiasis control programme, (iv) Madziwa and Goromonzi schistosomiasis control programmes, and (v) Plant-based molluscicides for schistosomiasis control.

### 3.1. Kariba Dam Schistosomiasis Control Programme

The schistosomiasis control programme for Kariba was initiated in 1967 after cases of the disease attended to at local health facilities increased significantly from 1963 when the lake filled for the first time. The control programme mainly focused on focal mollusciciding using niclosamid, and systematic screening and treatment of all residents. Shorelines were kept free of weeds particularly *Salvinia auriculata,* which was known to reintroduce snails in sprayed areas. The programme was funded by local companies and implemented by the Lake Kariba Area Coordinating Committee with technical backup from the Blair Research Laboratory, a disease control unit of the Ministry of Health. Routine snail surveys which informed what areas needed to be sprayed indicated that one area where the company had refused to participate in the control programme continued to harbor snails.  Table 1 shows compiled results of surveys conducted between 1967 and 2001. Although the systematic control activities were terminated in the late 1980s, assessments done after year 2000 [7] showed lower prevalence on the Zimbabwean side of Lake Kariba compared to the Zambian side, and the differences were attributed to a long history of schistosomiasis control activities on the Zimbabwean side and better water and sanitation facilities than on the Zambian side [7].

### 3.2. Hippo Valley Sugar Estates Schistosomiasis Control Programme

**Figure 3:**
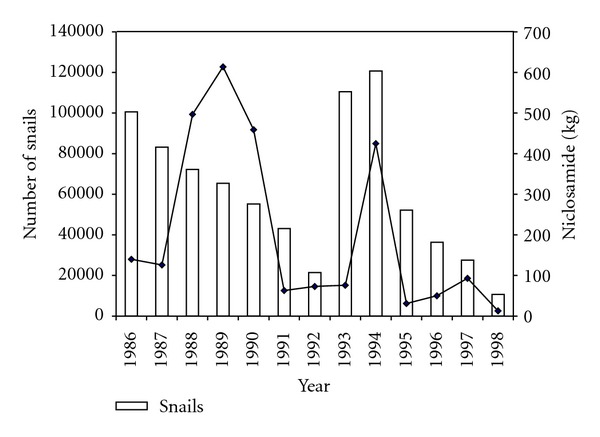
Snail population densities and quantities of niclosamide applied to reduce the snail numbers from 1986 to 1998 [9].

**Figure 4:**
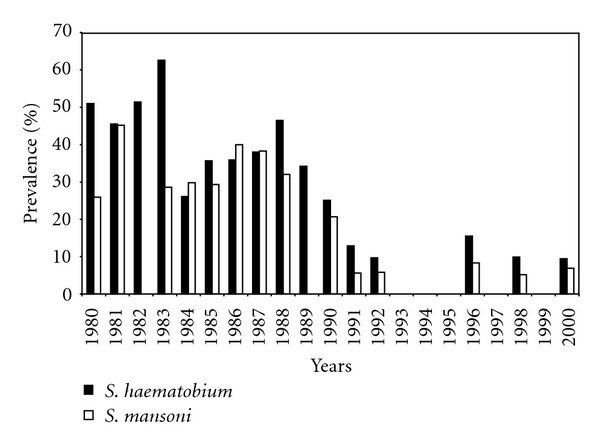
Prevalences of *Schistosoma haematobium* and *S. mansoni* among school children in Hippo Valley Estates for the period 1980–2000 [9].

The Hippo Valley Sugar Estates Schistosomiasis control programme was started in 1971 as a pilot project [17] that covered both the Hoppo Valley and Triangle Sugar Estates located in the south east lowveld region of Zimbabwe. The pilot project was later scaled up, and the programme placed greater emphasis on snail control using niclosamide and ducks as biological control agency. Alongside the snail control aspects, the programme had an annual chemotherapy component targeting school children and an intensive water and sanitation component. Assessments of efficacy of the control programme [9, 18] showed a significant decline in both prevalence and intensity over long periods and a sustained phase of prevalence below 10% (Figures 3 and 4). The success of the Hippo Valley story cannot be fully attributed to any one of the control strategies as each component made a significant contribution.

### 3.3. Mushandike Schistosomiasis Control Programme

The Mushandike project is a good example of a win-win intersectoral collaboration. The project was initiated in 1986 [19] with the objective to increase agricultural production of small-scale farmers through irrigation. Farmers were allocated farms ranging from 0.5 to 1.5 hectares, and irrigation was through siphoning water from tertiary canals onto the fields. The infield canals were fed by a 25 km main canal that made it necessary to have infield night storage ponds for smooth commanding of the fields.

At conceptualization and design of the irrigation scheme, there was consultation between health professionals interested in disease control and engineers responsible for designing the scheme. It was agreed that schistosomiasis was a potential health hazard that would impact negatively on crop production. Thus, the design was influenced such that the infield network of canals would all be lined in order to ensure fast movement of water to dislodge any snails present in the system and to avoid unnecessary water seepage. The in-field canal system included special features designed to flush snails (drop structures with stilling basins, special off takes, and duck bill weirs). Toilets constructed and arranged in a matrix system that ensured that people in the fields were at all times closer to a toilet than to a bush [20]. Water management was designed in such a way that canals in some irrigation blocks would be completely dry when not under irrigation, and only water needed for irrigation was released thus limiting end of field flooding. This was made possible by making sure that each block had one crop and, therefore, water demand would be the same. While the night storage ponds were undesirable, they could not be avoided from an engineering perspective but it was envisaged that proper operation of the night storage ponds would expose snails to predators during the draw down period. Furthermore the changing water levels would make the environment not conducive for snail colonization and establishment.

Monitoring of schistosomiasis conducted at Mushandike for a period of 5 years consistently showed higher levels of infection in the irrigation scheme where schistosomiasis control measures were not introduced (control farm) compared to the irrigation scheme where schistosomiasis control measures were introduced (intervention farms) [21]. Similar observations were made in a survey conducted 10 years after the project became operational (Table 2). Furthermore, a comparison of prevalences for 1989 to those obtained in 1999 showed that both *S. haematobium* and *S. mansoni *prevalences did not change significantly in villagers not attending school over a period of 10 years [16]. The prevalence of *S. haematobium* in the control farm was significantly higher than that of intervention farms implying that the engineering and environmental interventions may have contributed towards the difference.

Snail surveys conducted during the initial 5-year period and in 1991 also consistently showed high numbers of intermediate host snails present in the control farm than in the intervention farms. Furthermore, larger proportions of intermediate host snails collected in the control farm were infected with schistosome parasites compared to those collected in the intervention farms.

Although the costs involved in developing the “Mushandike Model” irrigation scheme are substantial, the Department of Irrigation in Zimbabwe adopted the model as the standard for all-small scale irrigation schemes. From a disease control perspective, the costs are justified as indicated by the incremental ratio of -$446 010.31 per 1% schistosomiasis prevalence, which meant that a saving of $446 010.31 per schistosomiasis prevalence of 1% was realized over a 10-year period [16].

### 3.4. Madziwa and Goromonzi Schistosomiasis Control Programmes

The Madziwa and Goromonzi schistosomiasis control projects were implemented in 1985–1989 [22] and 1994–1997 [23], respectively. Common to both projects were strong water and sanitation components, chemotherapy targeted to school children, and health education. The main differences in approaches used in the two intervention studies were that the Goromonzi health education component adopted the participatory health and hygiene education (PHHE) approach and mollusciciding was done once at the beginning of the project along the main stream in Madziwa while for Goromonzi only monitoring of sites for intermediate host snails was done in all major rivers and streams.

A 60% to 20% reduction in prevalence of *S. haematobium* infections among children aged 7–15 years was achieved at Madziwa. Furthermore, a 95% reduction in heavy infections among the targeted age group (7–15 years) was also achieved. Heavy infections were defined as greater than 50 *S. haematobium* eggs per 10 mL of urine or greater than 100 *S. mansoni* eggs per gram of faeces. In Goromonzi, prevalence of schistosomiasis among children aged 6–15 years declined from 20% to less than 5% for *S. mansoni* and from 40% to 10% *S. haematobium*.

 In both studies (Goromonzi and Madziwa), the differences in prevalence of infection between schools in intervention villages and schools in control villages were not significantly different and this was attributed to spill over of interventions largely because the villages were close to each other. Furthermore, infections increased to preintervention levels when chemotherapy was discontinued [23].

## 4. JICA Funded School Screening, Treatment and Education Programme

Upon a request by the Ministry of Health and Child Welfare (MOHCW), the Japanese International Cooperation Agency (JICA) partnered with the ministry to embark on a project with the following purposes: (i) to control specified infectious diseases such as schistosomiasis and malaria in the eight model districts and (ii) to formalize the National Schistosomiasis Control Policy based on the Project's experiences.

The project focused on the following 8 districts that became known as the model districts: Hurungwe, Mt Darwin, UMP, Lupane, Gokwe, Bulilimamangwe, Chipinge, and Mwenezi. School children in grade one (6 years) to grade 5 (10 years) were screened for schistosome infections and treated under a programme referred to as School Screening, Treatment and Education (SSTE) over a period of two years (1997–1999). Staff in131local health centres and at provincial and district level were trained on how to conduct SSTE. The trained staff with technical support from Blair Research Laboratory and JICA experts conducted SSTE in 497 out of the 631 schools in the model districts resulting in 85 578 out of the 102 000 children enrolled in the schools being screened and 99.4% of those found infected treated.

Prevalence and intensity of infection was significantly reduced over the two-year period, and a study conducted in one of the model districts (Mt Darwin) showed improved knowledge on schistosomiasis by school children but not a change in behavior [24], and no correlation between level of knowledge and infection rates was established. The draft policy document on schistosomiasis was adopted by model districts and therefore formed the basis for development of the final policy document worked on following the latest national survey [2].

### 4.1. Plant-Based Molluscicides for Schistosomiasis Control

Two plant-based molluscides (*Phytollacca dodecandra* and *Jatropha curcas*) have been studied with a view to use them in preference to the WHO recommended molluscicide, niclosamide. *Phytolacca dodecandra* has been studied in sufficient detail to justify its application in selected areas [25–30]. *Jatropha curcas* studies in Zimbabwe were only done in the laboratory [31] where the potency of the plant berries was demonstrated, showing that the unripe stage (green) of the berries was more potent than the ripe (yellow) and overripe stages (brown). The advantage of *J. curcas* over *P. dodecandra* is that the former has multipurposes (including potential for bio-fuel) and hence presents an incentive for farmers to grow it for financial benefits. However, the potency of a water extract of *J. curcas* is much lower (75 ppm) compared to that of *P. dodecandra *(10 ppm) implying that larger quantities of Jatropha berries would be required to sustain snail control activities.

Contrary to *J. curcas*, *P. dodecandra* has been extensively studied [25–30]. The variety of the plant that produces the most potent berries under the Zimbabwean agro-conditions was identified [26], and trials conducted along two natural streams showed that sites at which the molluscicide was applied was kept free from snail infestation for 7 months [28]. It was demonstrated under laboratory conditions that sublethal doses (<10 ppm) could be used to stop miracidia from successfully penetrating snail intermediate host snails for schistosomiasis [29]. The extent to which communities could be empowered to grow, harvest, process, and apply the molluscicide with minimum technical support has been described [31]. The results showed low level community participation due to, among other reasons, poor leadership, low economic value of the plant, inaccessible fields, and lack of tangible benefits. Despite these challenges, some districts adopted the use of the plant in the control programmes.

### 4.2. Biological Control Trials

Studies on exploring the possibilities of controlling intermediate host snails for schistosomiasis using a variety of biological agents have been conducted in Zimbabwe. The most studied biological agents tested include ducks, fish (*Sargochromis codringtonii*), and competitor snails (*Bulinus tropicus*).

#### 4.2.1. Ducks

Ducks were used in the Hippo Valley schistosomiasis control programme for many years as a supplement to application of niclosamide. The use of ducks was restricted to night storage ponds where a number of ducks would be allowed to swim around in one pond for 8 hours before being moved to another pond. While the ducks made significant impact in terms of reducing snail numbers in ponds there were several challenges faced with this intervention strategy. The costs associated with transportation of the ducks and looking after them to avoid poaching were high. Furthermore, the breeding and maintenance costs of the ducks were high as they were exotic species. In an effort to want to reduce costs of duck operations, semifield pond studies to investigate the potential of using indigenous ducks for snail control were conducted. The study concluded that there was potential for using indigenous ducks as biological snail control agents but further work needed to be done [32].

#### 4.2.2. Fish (*Sargochromis codringtonii*)

**Figure 5:**
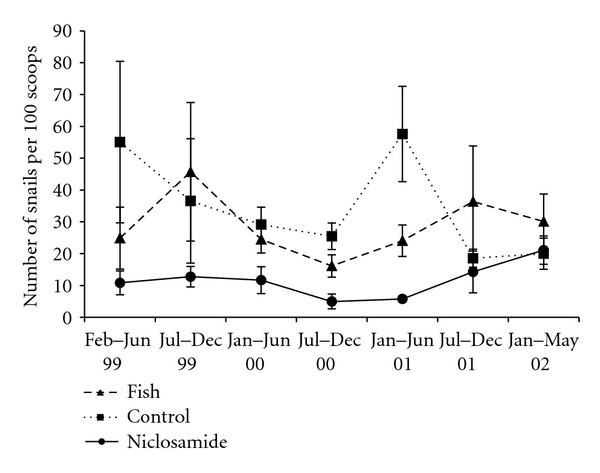
Average number of snails collected from night storage ponds from February 1999 to May 2002 [14].

Inspired by the observations that overfishing of cichlid fish in Lake Malawi shorelines resulted in increased numbers of snails and increased cases of schistosomiasis [33] and studies conducted in Lake Kariba [34], comprehensive studies aimed at testing the potential of using an indigenous cichlid to Zimbabwe, *S. codringtoni,* as a biological agent for snail control were conducted. Laboratory studies [35, 36] demonstrated the snail eating tendencies of *S. codringtonii* and the interactions of snails (prey) and fish (predator) under aquaria conditions. Enclosure [37], and exclosure [38] showed the effects of snail predation on *S. codringtonii* under different treatments: with vegetation, in combination with fish herbivore (*Tilapia rendalli*); with a wider choice of snails (both pulmonates and prosobranchs). The results showed that pulmonates but not necessarily intermediate host snails were preferred by *S. codringronii* and that vegetation provided refugia for snails against the predator fish. However, the combination of *S. codringtonii* and *T. rendalli* was not desirable as the later was attacked by the former. Field studies conducted in night storage ponds [37] further demonstrated the potential use of *S. codringtonii* as a biological agent but the results were not conclusive as the monitoring period was short (Figure 5). It was, however, evident that *S. codringtoni,* which is mainly found in Lake Kariba, could acclimatize to small ponds (100 m × 100 × 1–1.5 m depth) in the Lowveld of Zimbabwe. The predator-prey interactions of *S. codringtonii* and snails have also been studied [14, 38–41].

The extensive studies on *S. codringtonii* as biological agent for controlling intermediate host snails for schistosomiasis provide convincing evidence to justify use of the fish in appropriate settings like night storage ponds in irrigation schemes where the fish could be a good source of protein and serve as a snail control agent.

#### 4.2.3. Competitor Snails (*Bulinus tropicus*)

Motivated by the observation that *B. globosus* (intermediate host snail for schistosomiasis) and *B. tropicus* (non-intermediate host snail for schistosomiasis) do not share the same niche although they share similar habitats, studies aimed at investing the potential of using *B. tropicus* as a competitor snail of *B. globosus* with the ultimate goal of controlling schistosomiasis were conducted [42, 43]. Laboratory and quasifield studies showed a significant reduction in reproductivity of *B. globosus* in the presence of *B. tropicus* and evidence of *B. tropicus* preying on *B. globosus* eggs [42]. However, further enclosure studies [43] did not show any significant effect of *B. tropicus* on *B. globosus* population density suggesting the competition between the two snail species was not important control of schistosomiasis.

## 5. Discussion

Understanding the life cycle of a parasite and the epidemiology of the disease caused by the parasite are fundamental to disease control. Following the first description of schistosomiasis in man by Theodor Bilharz in 1851, the life cycle was studied and described [44]. The transmission dynamics of the disease in Zimbabwe has been well documented [3–11, 17, 18, 22]. There are four broad interventions that can be made to disrupt the life cycle of the parasite and hence its transmission; (1) treatment of infected individuals to reduce, and remove morbidity, reduce mortality and reduce contamination of the environment with schistosome parasite eggs, (2) providing communities with adequate, appropriate sanitation to reduce environmental contamination and hence minimize the chances of miracidia finding and penetrating the intermediate host snails, (3) snail control to minimize the chances of miracidia finding an appropriate intermediate host and therefore significantly reducing the number of cercariae available for infecting people at water contact sites, and (4) provision of adequate and accessible safe water to reduce the chances of people getting in contact with water that may be infested with cercariae and hence limits the chances of cercaria locating the human host and infect them in its limited life span. All the aforementioned possible interventions have been studied in detail globally and at local level, and it is appreciated that simultaneous implementation of all the measures may not be cost effective. Hence, treatment has been prioritized as it reduces early and late life morbidity and mortality, and eventually reduces the force of transmission by reducing contamination.

The experiences in treatment of infected individuals reviewed in this paper for Zimbabwe clearly show that scaling up this strategy will not be a difficult task. The Hippo Valley and Mushandike [8, 18, 19] experiences can inform the treatment strategy for communities in both large- and small-scale irrigation schemes. The local level capacity developed during the SSTE JICA programme and during the 1992 and 2010 surveys [2, 3] is an asset in rolling out a national control programme that is school based in line with WHO guidelines [12]. Thus, with adequate government commitment to resource the control programme and donor/partner support particularly in the area of drug procurement, success in schistosomiasis control in Zimbabwe can be achieved.

While WHO guidelines [12] do not negate other key schistosomiasis control measures described in this paper, it is clear that greater emphasis is placed on treatment. However, at country level the experience gained in the other nontreatment measures cannot be ignored. The use of niclosamide for control of intermediate host snails is not practical for application in communal areas and other poorly resourced communities because of logistical, financial, and environmental reasons. However, this is a strategy that can continue to be promoted in the lowveld where it has been proven to be successful. Given the huge research investment made on *P. dodecandra* and the positive results obtained in field trials [26–31], there is justification in scaling up this intervention particularly in communal areas and small-scale irrigation schemes. However, the challenges associated with its application in communal areas [31] will need to be addressed and close monitoring of environmental impacts will need to be done. Given the current drive towards use of *J. curcas* for biofuel, there is a need to conduct further research on possible use of the plant as molluscicide as there are likely to be better incentives to grow *J. curcas* than to grow *P. dodecandra*. In general, the mollusciciding strategy should be focal in nature to minimize costs and environmental impacts with the exception of irrigation systems where there is a need to treat the complete canal network.

The challenges associated with biological control in general are known [45]. Predator-prey interactions will lead to some equilibrium and that equilibrium threshold may not be adequate for purposes of controlling the prey to the desired level. This is particularly so in the case where *B. tropicus* may needs to be used as a competitor for *B. globosus. *The inconclusive results as reviewed in this paper show that this may not be an area to make further investments. However, the potential use of indigenous ducks and *S. codringtonii* need to be seriously considered particularly in irrigation ponds but also in communal ponds or small dams. This is because these biological agents have broad spectrum diets, which will allow them to switch to another less preferred prey if the preferred one is absent. Furthermore, the agents may contribute significantly to community protein requirements. Ducks would be provided with alternative feeds, and a possibility for supplementing fish with inexpensive feeds can be explored once the snail numbers have reached too low numbers to maintain a reasonable population size of the fish. Dietary shift studies will need to be carried out to establish if there might not be a permanent shift of diet from snails to other food items.

Zimbabwe is better positioned to apply the ordinarily expensive interventions of water and sanitation in schistosomiasis control as home grown technologies have been developed and tested in the field [46]. Furthermore, promotion of water and sanitation interventions will impact on more than one neglected tropical disease [47] and will generally improve the quality of human life particularly in rural settings. The latter reason is likely to garner support of NGOs and other international organizations keen on improving rural community health and livelihoods.

In conclusion, I recommend that Zimbabwe should adopt the WHO recommended strategy for controlling schistosomiasis and in doing so should seriously consider some of the measures proven to be effective at local level but are less emphasized in the guidelines. Specifically, the snail aspects should be seriously considered to avoid a situation where the only safe efficacious drug for schistosomiasis, praziquantel, may one day be compromised by parasite resistance and result in an outbreak. Besides, snail control complement well the treatment strategy. In an effort to achieve the objective of controlling schistosomiasis there is a need to ensure that the policy for control is passed by parliament and guidelines to operationalize the policy are developed. The huge human resource base in the area of schistosomiasis developed since 1990 should be fully utilized to achieve control and move towards elimination. Since 1990, fifteen staff were trained to Ph.D. level and more than 20 technicians were trained most of them to M. S. level. While a small proportion have passed-on, the remaining “Zimbabwe Schistosomiasis Scientists” spread in the southern Africa region and overseas are very committed to the cause of control and are currently supporting in-country initiatives.

## Figures and Tables

**Table 1:** Results of schistosomiasis surveys conducted around Kariba town [15].

Year	Area	Population category	Prevalence of *S. haematobium* (%)	Prevalence of *S. mansoni* (%)
1967	Kariba Town	Adult workers	13.3	8.6
1979	Mahombekombe	School children	54.6	68.0
	Nyamhunga	School children	48.0	64.0
1984	All government departments and industries	Adult employees of all government departments and private sector	9.4	14.3
1985	All government departments and industries	Adult employees of all government departments and private sector	4.8	8.1
1986	All government departments and industries	Adult employees of all government departments and private sector	8.4	10.5
2001	Mahombekombe,	School children	9.0	2.5
	Nyamhunga and Charara	Subsistence fishermen	7.3	12.5
		Commercial fishermen	0	26.3

**Table 2:** Showing the prevalence of *S. haematobium* in the study population [16].

	Intervention villages					Control village
	Village 12	Village13	Village 14	Village15	Total	Chikore
*S. haematobium*						
Number positive	5	5	4	8	22	10
Number negative	98	58	42	57	255	69
Prevalence	4.9%	7.9%	8.7%	12.3	7.9%	12.7%
*S. mansoni*						
Number positive	0	3	3	3	9	No data
Number negative	103	60	43	62	268	No data
Prevalence	0	4.8	6.5	4.6	3.4%	No data
